# *Plasmodium vivax*: paroxysm-associated lipids mediate leukocyte aggregation

**DOI:** 10.1186/1475-2875-6-62

**Published:** 2007-05-22

**Authors:** Nadira Karunaweera, Deepani Wanasekara, Vishvanath Chandrasekharan, Kamini Mendis, Richard Carter

**Affiliations:** 1 Malaria Research Unit, Department of Parasitology, Faculty of Medicine, University of Colombo,, P.O. Box 271, Kynsey Road, Colombo 08, Sri Lanka; 2 Department of Biochemistry, Faculty of Medicine, University of Colombo, Sri Lanka; 3 Institute of Immunology and Infection Research, School of Biological Sciences, University of Edinburgh, Edinburgh, UK

## Abstract

**Background:**

Paroxysms are recurrent febrile episodes, characteristic of *Plasmodium vivax *infections, which coincide with the rupture of schizont-infected erythrocytes in the patients' circulation. The present study describes the formation of prominent aggregates of leukocytes *in vitro *in the presence of parasite and host factors released during paroxysms.

**Methods:**

Whole blood cells from uninfected malaria-naïve donors were incubated with plasma taken during a paroxysm or normal human plasma as a control and cell smears were observed under the microscope for the presence of leukocyte aggregates. Plasma factors involved in mediating the leukocyte aggregation were identified using immune depletion and reconstitution experiments. Furthermore, biochemical characterization was carried out to determine the chemical nature of the active moieties in plasma present during paroxysms.

**Results:**

Leukocyte aggregates were seen exclusively when cells were incubated in plasma collected during a paroxysm. Immune depletion and reconstitution experiments revealed that the host cytokines TNF-alpha, GM-CSF, IL-6 and IL-10 and two lipid fractions of paroxysm plasma comprise the necessary and sufficient mediators of this phenomenon. The two lipid components of the paroxysm plasmas speculated to be of putative parasite origin, were a phospholipid-containing fraction and another containing cholesterol and triglycerides. The phospholipid fraction was dependent upon the presence of cytokines for its activity unlike the cholesterol/triglyceride-containing fraction which in the absence of added cytokines was much more active than the phospholipids fraction. The biological activity of the paroxysm plasmas from non-immune patients who presented with acute *P. vivax *infections was neutralized by immune sera raised against schizont extracts of either *P. vivax *or *Plasmodium falciparum*. However, immune sera against *P. vivax *were more effective than that against *P. falciparum *indicating that the parasite activity involved may be antigenically at least partially parasite species-specific.

**Conclusion:**

Leukocyte aggregation was identified as associated with paroxysms in *P. vivax *infections. This phenomenon is mediated by plasma factors including host-derived cytokines and lipids of putative parasite origin. The characteristics of the phospholipid fraction in paroxysm plasma are congruent with those of the parasite-derived, TNF-inducing GPI moieties described by others. The more active cholesterol/triglyceride(s), however, represent a novel malarial toxin, which is a new class of biologically active lipid associated with the paroxysm of *P. vivax *malaria.

## Background

The most characteristic presentations of acute *Plasmodium vivax *infections are the periodic episodes of fever with chills and rigors, which follow the rupture of schizont-infected erythrocytes in the patients' circulation. Previous investigations have been made on events associated with paroxysms in *P. vivax *infections including the inactivation of sexual stages of the parasites (gametocytes) in the presence of plasma taken at the time of a paroxysm [1,2]. These studies have demonstrated the roles in this process of the human cytokines TNF-α, GM-CSF and IL-2 together with parasite products. Previous studies also demonstrated the prominent rise and fall of TNFα – levels which corresponds very closely to the rise and fall of fever during the paroxysms [3] and of the transient appearance of elevated numbers of gamma/delta T cells in the peripheral circulation at the time of these events [4].

In the present study, the aggregation of peripheral blood white cells was investigated in the presence of plasmas taken at the time of a *P. vivax *paroxysm. These cell aggregations were formed only in the presence of paroxysm plasma and not in the presence of plasma from before or after a paroxysm, or from a healthy donor. Further, this paroxysm plasma-mediated cell aggregation phenomenon was exploited as an assay to identify and characterize biologically active mediators of host and parasite origin during the paroxysms of *P. vivax *malaria. This assay enabled preliminary chemical and physical-chemical characterization of parasite-derived products present and active during a *P. vivax *paroxysm.

## Methods

### Patients

Two study groups were included. They were patients with *P. vivax *infections from two localities in Sri Lanka. Those from the first were adult residents of malaria-non-endemic regions attending the National Hospital of Sri Lanka, in Colombo. These patients were residents of Colombo and its suburbs where there is no local transmission of malaria; they had acquired their infections following travel to a malaria-endemic area of Sri Lanka. These patients (except six patients who had one past infection) had no previous recorded malarial infections and were immunologically naïve (non-immune) with respect to malaria.

The second group of patients comprised adults in a *P. vivax*-endemic region of Sri Lanka at Kataragama [5]. The patients were similarly diagnosed as having *P. vivax *infections by blood smear examination. All these individuals had experienced several previous *P. vivax *infections (8–28 infections; median 18), as revealed during verbal interview and also based on the case records. The clinical symptoms of paroxysms were invariably milder in these patients from Kataragama (most did not experience rigors) than in non-immune patients from Colombo; these patients were considered as clinically semi-immune with respect to malaria [6].

### Control groups

Two age and sex matched control groups were included. One group consisted of healthy volunteers residing in areas with no local malaria transmission and with no past history of malaria infections. The second control group included healthy individuals resident in Kataragama (a malaria-endemic area). All these individuals were blood film-negative for malaria parasites.

### Cell samples

Five ml of blood samples were collected from apparently healthy individuals residing in Colombo who were blood film-negative for malarial parasites and who did not give a past history of malaria. Blood was collected (and diluted) into a solution containing Tris-1.2 mg/ml (Biorad), NaCl – 8.2 mg/ml (Sigma) and Glucose – 1.8 mg/ml (Sigma) at pH 7.4 at 10% concentration [2]. Whole blood cells were separated by centrifugation at a speed of 800 × G for 10 minutes. The supernatant containing the diluted plasma was discarded. The upper 40% of the cell pellet, mainly consisting of white blood cells, was pipetted out. This was mixed gently to make a homogenous cell suspension.

### Plasma samples

Following identification of the infection by blood smear, informed volunteers completed a single paroxysm without drug treatment. The time of paroxysms was predetermined by the increasing oral temperature (above 37.8°C) and the presence of chills in most cases and supported by examination of the blood smears made from these patients. From the onset to the end of fever paroxysms lasted 4 – 5 hours. Two milliliters of blood were collected for plasma prior to the onset of paroxysm (pre-paroxysm plasma samples) into 0.1% EDTA containing a protease inhibitor (aprotinin; 0.6 trypsin inhibitor U/ml). A second set of samples (paroxysm plasma) was collected at, or within one hour of, the peak of a rigor and a third set, four to six hours after the fever peak (post-paroxysm plasma). Serum samples were collected from *P.vivax-*infected patients, four to six weeks after drug cure (convalescent serum).

Another set of plasma samples was collected from fever patients admitted to the National Hospital of Sri Lanka with oral temperatures above 37.8°C and blood film-negative for malarial parasites when tested at least twice on consecutive days (non-malarial fever plasma).

Control plasma samples were collected from healthy volunteers (normal human plasma).

### Leukocyte aggregation

Whole blood cells were obtained from blood samples collected from apparently healthy individuals. 80 μl of cell suspension rich in white blood cells (obtained as described under cell samples), was pipetted into 800 μl of RPMI 1640 [1] in a 24-well tissue culture plate (Flow Laboratories, UK) and 200 μl of test (paroxysm plasma, pre-paroxysm plasma, post-paroxysm plasma, non-malarial fever plasma or plasma from clinically semi-immune endemic patients) or control plasma samples (normal human plasma) were added into the cell suspension. The culture plate was incubated for 3 hours at 37°C in an incubator. The supernatant was discarded and from the remaining cell pellet 40 μl was pipetted out and dispersed evenly on a microscopic slide, in a circle of 1.5 cm diameter using a template. These blood smears were air dried, stained with Giemsa stain and examined under 400× magnification.

### Cell aggregation index

Blood smears were prepared from cultures containing cells from healthy individuals incubated with test or control plasma samples as described above. Nucleated cell aggregates containing five or more cells and the number of un-clumped cells (that included single nucleated cells and clumps of less than five cells) were counted in 100 microscopic fields and the total number of the nucleated cells in these fields were also noted.

Based on these counts, a 'cell aggregation index' (CAI), indicative of the ability of a particular plasma sample to induce aggregation of white blood cells, was calculated as follows:

CAI = ([Total no. of white blood cells/(No. of clumps + no. of un-clumped white cells)] - 1), in 100 microscopic fields at 400× magnification

% Relative Cell Aggregation Index (RCAI) = [CAI of test sample/CAI of paroxysm plasma] × 100

### Characterization of cell types involved in inducing cell aggregation

#### Monocytes

Monocyte depletion was done using two methods:

##### Method A

80 μl of cell suspension was diluted with 80 μl of a buffer solution (containing Phosphate Buffered Solution/Foetal Calf Serum at 1:1 ratio). This cell suspension was added (at1:1 ratio) to a suspension of magnetic polystyrene beads coated with a primary monoclonal antibody specific for the CD14 membrane antigen (Dynabeads M-450-CD14, Dynal A.S., Oslo, Norway). This mixture was then incubated for twenty minutes at 4°C. After incubation, the tube containing the cell suspension was placed in the magnetic particle concentrator (Dynal, MPC-1, Dynal A.S. Oslo, Norway) which has a magnetic field to attract the antibody coated Dynabeads together with the attached cells. The remaining cells were pipetted out and this step was repeated five times. The remaining cell suspension (depleted of CD14 monocytes) was used for the relevant experiments. Absence of monocytes from the cell suspension was confirmed using blood smears which were made on glass slides, stained and examined under the microscope.

##### Method B

80 μl of cell suspension was depleted of monocytes using the plate-adherent method described previously [7]. Briefly, the white cell-rich cell suspension was incubated at 37°C for 30 minutes in a polystyrene petri dish (size: 35 × 10 mm, Becton Dickinson and Co.) at 10% cell to liquid volume in RPMI 1640. Then the supernatant containing the non-adherent cells was carefully removed and the cells were spun down.

Monocyte-depleted cells thus obtained by each method were resuspended and used in the cell aggregation assay as described above.

#### Monocyte reconstitution

For reconstitution experiments, a preparation of mononuclear cells was obtained from whole blood by sodium metrizoate density gradient centrifugation (Lymphoprep, Norway) according to standard procedure [1]. The mononuclear cells were resuspended in RPMI 1640 pH 7.4 at 10% concentration and introduced into polystyrene petri dishes (size: 35 × 10 mm, Becton Dickinson and Co.). 1 ml volumes at 2 × 10^7^/ml concentrations were incubated at 37°C for half an hour [7]. After incubation, the cell suspension was pipetted out. The cells adhered on the surface of the plate were scraped out with the rubber end of a 1 ml syringe plunger and a cell count was taken using a haemocytometer. Cells thus obtained (monocytes) were used for reconstitution experiments at a concentration of 6 × 10^6 ^cells per well.

#### T cells

T cell depletion:T cells were depleted from 80 μl of cell suspension by following the same procedures as adopted in the case of macrophages, using Dynabeads coated with antibodies specific for CD2 membrane antigen (Dynabeads-CD2, Dynal A.S., Oslo, Norway). The remaining (T cell-depleted) cell suspension was used for relevant experiments.

### Identification of factors mediating cell aggregation

#### (i) Parasite factors

##### Effect of neutralization of parasite factors on paroxysm plasma-induced cell aggregation

Serum containing anti-parasite antibodies i.e. hyper-immune rabbit serum raised against freeze-thawed extracts of either *P. vivax *or *P. falciparum *[2] or with human convalescent serum was pre-incubated with paroxysm plasma at 1:1 of concentration for 30 minutes before incubating with un-primed whole blood cells collected from healthy individuals.

##### Effect of re-constitution of parasite factors in normal human plasma treated with freeze-thawed *P. vivax *schizont extracts

Normal human plasma obtained from healthy individuals were reconstituted with freeze-thawed extracts of 5 × 10^6 ^*P. vivax *schizonts per ml or 5 × 10^6 ^of uninfected red blood cells per ml as a control prepared as previously described [1]. This re-constituted plasma was incubated with unprimed whole blood cells of healthy individuals.

As a control, normal human plasma was reconstituted with *E. coli *lipopolysaccharide (LPS) at a final concentration of 2 μg/ml and tested in cell aggregation assay as previously described [1].

#### (ii) Host factors

##### Cytokines

Involvement of cytokines in the mediation of cell aggregation by paroxysm plasma was investigated by their neutralisation with anti-cytokine antibodies and reconstitution with the addition of recombinant human cytokines. The optimum concentrations of anti-cytokine antibodies and recombinant cytokines required to overcome the neutralization imposed by the antibodies were determined based on experiments using dilution series of these, using the same principles and methodologies adopted in previous studies [8].

#### Depletion and reconstitution of plasmas with specific components

##### Depletion experiments

Paroxysm plasmas were pre-incubated at 37°C for 30 minutes with the following immune reagents, singly or in combination, before assessing the effect on paroxysm plasma-induced cell aggregation.

(i) Rabbit polyclonal antibodies (IgG) against human IL1-α [0.014 μg/ml], IL1-β [0.075 μg/ml], IL-2 [1.5 μg/ml], IL-3 [5 μg/ml], IL-4 [0.125 μg/ml], IL-6 [0.1 μg/ml], IL-10 [7.5 μg/ml], IFNγ [5 μg/ml], TNF-α [0.04 μg/ml], TNF-α [0.05 μg/ml] and GM-CSF [5 μg/ml] (R&D systems, UK).

##### Reconstitution experiments

Recombinant human cytokines (IL1-α [5 pg/ml ], IL1-β [7.5 pg/ml], IL-2 [0.375 ng/ml], IL-3 [0.25 ng/ml], IL-4 [0.125 ng/ml], IL-6 [0.5 ng/ml], IL-10 [0.75 ng/ml]), IFNγ [1.15 ng/ml ], TNF-α [0.05 ng/ml], TNF-α [0.03 ng/ml] and GM-CSF [0.05 ng/ml] were added to cytokine-depleted paroxysm plasma to confirm the involvement of cytokines in paroxysm plasma-induced cell aggregation. Recombinant human cytokines were also added to normal human plasma or post paroxysm plasma in other experiments. Freeze-thawed extracts of 5 × 10^6^/ml *P. vivax *schizonts or 5 × 10^6^/ml of uninfected red blood cells as a control (prepared as previously described, [1]) were added to plasmas in relevant experiments.

The physical/chemical characterization of plasma factors of putative parasite origin that mediate leukocyte aggregation.

##### Heating

Paroxysm plasma and normal human plasma, as a control, were heated in 1 ml volumes in a water bath at 60°C, 80°C or 100°C for five minutes before testing in the cell aggregation assay. The heat-treated fraction of paroxysm plasma, was reconstituted either with an extract of *P. vivax *schizonts or with recombinant human cytokines (rh TNF-α, rh GM-CSF, rh IL-10, rh IL-6) and tested for cell aggregation.

##### Ultra-centrifugation

Paroxysm plasma and normal human plasma were centrifuged in 1 ml volumes at 180,000 × G for 15 minutes at 4°C. Two layers were formed. The thin opaque, whitish upper layer was removed in 100 to 200 ul. The remaining liquid was yellowish but clear and was retained as a single fraction. The two fractions were tested separately and reconstituted with recombinant human cytokines (rh TNF-α, GM-CSF, IL-10, IL-6) or with *P. vivax *schizont extract and re-tested in the cell aggregation assay. The separated fractions were also tested for lipids (standard Sudan III test) [9].

##### Filtration

1 ml samples of paroxysm plasma or normal human plasma were passed through 0.45 μm millipore filters (Flow laboratories, UK) and the filtrates were tested in the cell aggregation assay. The filtrates were re-tested in the cell aggregation assay following reconstitution with recombinant human cytokines (rh TNF-α, GM-CSF, IL-6, IL-10) or *P. vivax *schizont extracts.

##### Extraction and fractionation of lipids

1 ml samples of paroxysm plasma, or 1 ml of normal human plasma (NHP) as a control, were heated at 100°C for five minutes in a water bath. Freeze-thawed extracts of *P. vivax *schizonts at 5 × 10^6 ^schizonts in 1 ml of NHP were also heat-treated in the same way. The following extractions were then carried out, all at room temperature, using standard biochemical techniques [10-12]. Briefly, each of the heat-treated plasma samples was extracted first with 1 ml of acetone, centrifuged for 10 seconds at × 15,000 × G and the supernatant retained as fraction 1 (cholesterol, triglycerides); the pellet was extracted with 1 ml of 95% ethanol, spun as before and the supernatant retained as fraction 2 (lecithin); the pellet was extracted in 1 ml of petroleum-ether, spun as before and the supernatant retained as fraction 3 (composition unidentified). The pellet obtained, and half of the retained fraction 3, were added together and extracted with 1 ml of diethyl-ether, spun as before, and the supernatant retained as fraction 4 (phospholipids) and the pellet as fraction 5 (sphingolipids). Each of these fractions was evaporated to dryness by flushing with nitrogen gas. The residues were dissolved in 1 ml either of normal human plasma or of normal human plasma reconstituted with recombinant human cytokines (rh TNF-α, GM-CSF, IL-6 & IL-10) as described above and then tested in the cell aggregation assay.

### Statistical analysis

As the data dispersion was found to be not normal, Mann-Whitney U test was applied to compare two groups and Kruskal – Wallis test was applied when there were more than two groups to be compared and the post hoc significance was analysed. Median values were given with inter-quartiles (as 25%–75% percentiles). (statistical software package – SPSS 10.0 for Windows).

### Ethical clearance

All aspects of the study were approved by the Ethical review committee of Faculty of Medicine, University of Colombo, Sri Lanka. Informed-written consent was obtained from all participants.

## Results

### Aggregation of white blood cells from healthy donors in the presence of paroxysm plasma from acute *P. vivax*-infected patients

**Figure 1:**
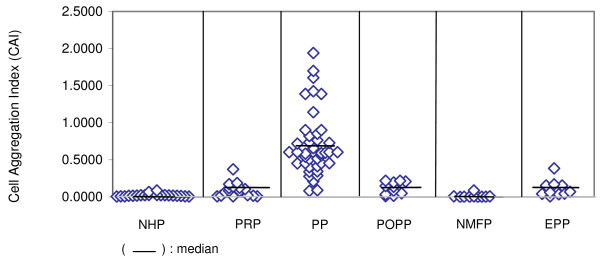
**Cell aggregation-inducing effect of plasma samples**. Blood samples were collected for plasma before, during and after paroxysm from non-immune *Plasmodium vivax*-infected patients, healthy individuals, non-malarial fever patients and from malaria patients resident in an endemic area (malaria semi-immune individuals); plasmas from these blood samples were tested for their ability to induce white blood cell aggregation *in vitro*. (p < 0.001: Kruskal Wallis test; p values of post hoc significance (Mann Whitney U test) are indicated in the text). NHP: Normal Human Plasma; PRP: Pre Paroxysm plasma; PP: Paroxysm Plasma; POPP: Post Paroxysm Plasma; NMFP: Non Malarial Fever Plasma; EPP: Endemic Paroxysm Plasma

Striking changes in the distribution of nucleated cells were observed between smears made from cultures in which whole peripheral blood cells from healthy individuals were incubated in the presence of paroxysm plasma from acute *P. vivax*-infected patients when compared with cultures incubated in the presence of normal human plasma. In smears made from cultures with normal plasma (NHP), most of the nucleated white blood cells were distributed in the smear either as single cells or in small aggregates of not more than 2–3 cells per cluster. In contrast, in blood smears of cultures containing paroxysm plasma (PP), obvious, large aggregations of nucleated white blood cells were seen, with a significantly high cell aggregation index (CAI) [Figure 1: (p < 0.001 when compared with NHP)].

The aggregates were composed predominantly of neutrophils (80% – 90%), monocytes and lymphocytes. They varied in size, ranging from five to100 cells with an average of 20. Marked aggregations of white blood cells with high cell aggregation indices were observed in the presence of all paroxysm plasma collected from non-immune patients who presented with acute *P. vivax *infections (Figure 1; PP).

There were very few cell aggregates seen in smears made from cultures containing pre-paroxysm plasma with resultant low cell aggregation indices (Figure 1; PRP); p < 0.001 relative to paroxysm plasma) or post-paroxysm plasma (Figure 1; POPP) (p < 0.001 relative to paroxysm plasma). These results demonstrate a tight coincidence in timing between the period of prominent clinical symptoms during paroxysms in *P. vivax *infections and the presence of plasma mediators that induce the aggregation of white blood cells *in vitro*.

The robustness (repeatability) of this bioassay system was tested by calculating the co-efficient of variation of the CAI for different PP samples (n = 21) and the same PP samples in repeated experiments (n = 6). The co-efficient of variation of the CAI ranged between 0 and 10% (except 3 values which were between 15% and 50%).

### The absence of the leukocyte aggregation-inducing factors in paroxysm plasma from semi-immune *P. vivax *patients and evidence for serum-mediated immunity against parasite factors/"toxins"

In contrast to the induction of leukocyte aggregation by paroxysm plasma from the clinically non-immune patients from Colombo, plasmas collected during paroxysm from age-matched, semi-immune, acutely infected *P. vivax *patients resident in Kataragama, an endemic area of Sri Lanka, had little such effect (Figure 1; EPP) (p < 0.001 relative to paroxysm plasmas from non-immune patients). These findings indicated an association between the absence of the cell aggregation-mediating activity in infection plasma and tolerance to the symptoms of paroxysms during malarial infection.

Convalescent serum collected following recovery from a *P. vivax *infection from these semi-immune endemic residents when pre-incubated with PP reduced the aggregation index of non-immune paroxysm plasma by 83% (Table 1: Expt.15 cf Expt.2; p < 0.05). Together with evidence that parasite products released during schizogony are active mediators of cell aggregation *in vitro *(see below), this finding suggests that clinical immunity may be associated with the presence of antibodies against these active parasite products.

**Table 1:** Effect of plasma samples, cytokines and parasite products in the white blood cell aggregation assay

**Expt. No.**	**Sample**	**% RCAI (Median)**	**25%**	**75%**
1	NHP	5.80	1.6	7.3
2	PP	100	100.0	100.0
3	POPP	20.90	5.6	28.4
4	NHP+cytokines	17.20	6.7	23.5
5	NHP+PvAg	18.60	4.5	20.8
6	NHP+PvAg+ cytokines	88.50	79.8	92.4
7	NHP+RBC Ag+ cytokines	10.00	2.4	12.9
8	POPP+cytokines	18.30	7.5	22.3
9	POPP+PvAg	16.40	9.4	18.1
10	POPP+PvAg+ cytokines	77.10	64.7	79.8
11	NHP+LPS	6.10	1.9	7.2
12	NHP+LPS+cytokines	16.10	6.9	18.6
13	PP + IRS-Pv	24.90	15.6	28.4
14	PP + IRS-Pf	54.40	48.6	65.4
15	PP + CHS	17.00	14.2	19.5

% RCAI: % Relative Cell Aggregation Index

25%–75%: 25th and 75th percentiles

Normal human plasmas (NHP) and post paroxysm plasmas (POPP) were reconstituted with freeze-thawed extracts of *P. vivax *schizonts (Pv Ag), normal red cell extracts (RBC Ag), recombinant human cytokines TNF-∝, GM-CSF, IL-6 and IL-10 (cytokines) or E. coli lipopolysaccharide (LPS). *P. vivax *paroxysm plasma (PP) was pre-incubated with rabbit serum raised against *P. vivax *schizonts (IRS-Pv), with rabbit serum raised against *P. falciparum *schizonts (IRS-Pf) or with convalescent human serum (CHS) collected 4 – 6 weeks after drug cure of a *P. vivax *infection, from semi-immune individuals resident in a malaria-endemic area prior to being tested in the cell aggregation assay. The table provides the median values together with the interquartile range (25%–75%).

### The absence of the leukocyte aggregation inducing factors in plasmas of non-malarial fever patients

Plasma samples which were obtained from febrile patients attending the National Hospital of Sri Lanka in Colombo, who were blood film-negative for malarial parasites (NMFP) did not cause any significant aggregation of white blood cells when compared with paroxysm plasma (Figure 1; NMFP; p < 0.001 relative to paroxysm plasma) indicating that the active factors present in paroxysm plasma were specific to malarial infections, though it remains a possibility that synchronised schizongony that operates prior to a paroxysm could trigger agent(s) that might be more concentrated in malarial plasma as opposed to in plasma from other febrile infections.

### The effects of the removal of monocytes or T lymphocytes on the aggregation of white blood cells mediated by paroxysm plasma from acute *P. vivax*-infected patients

**Figure 2:**
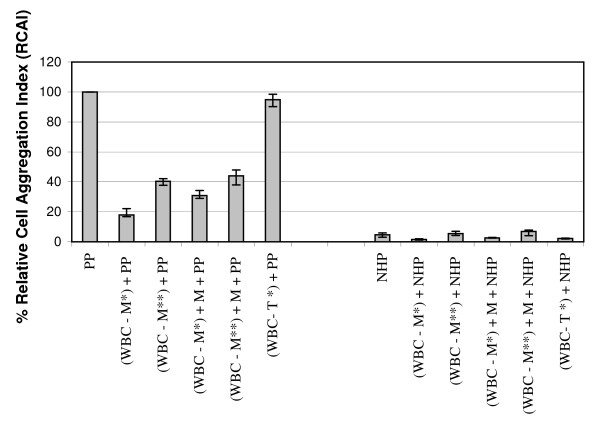
**Role of monocytes and lymphocytes in cell aggregation induced by *Plasmodium vivax *paroxysm plasma**. White blood cell suspensions were depleted of monocytes or lymphocytes prior to incubation with paroxysm plasma samples (n = 6) in the cell aggregation with or without reconstitution with these cell types. The bars indicate the median values of the data series together with the interquartile range (25%–75%). PP: paroxysm plasma; NHP: normal human plasma; WBC: white blood cells; T*: T cells depleted by Dynabeads (anti CD2), M *: monocytes depleted by Dynabeads (anti CD14), M**: monocytes depleted by panning method; M: monocytes recovered from panning method.

The degree of leukocyte aggregation mediated by paroxysm plasma from acute *P. vivax*-infected patients was markedly reduced when monocytes were depleted from the leukocyte suspension prior to incubation with paroxysm plasma (p < 0.05 relative to that with undepleted white blood cells) and was restored to some extent by reconstitution of the cell suspension with monocytes (p > 0.05 relative to that with undepleted white blood cells – Figure 2). No similar reduction in white cell aggregation was seen when T lymphocytes were depleted from the cell suspension (p > 0.05 relative to undepleted white blood cells). The findings indicate a likely role of monocytes in the formation of cell aggregates.

### Cytokines and probable parasite products are the active mediators of the paroxysm plasma-induced leukocyte aggregation phenomenon

**Figure 3:**
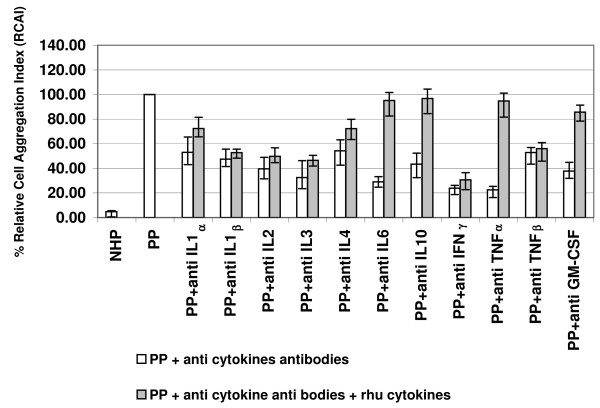
**The effect of cytokines in white blood cell aggregation mediated by *Plasmodium vivax *paroxysm plasma**. Plasmas obtained from non-immune *Plasmodium vivax *– infected patients (n = 6) during a paroxysm (PP) were tested in the cell aggregation assay following depletion and then reconstitution of that cytokine. The untreated paroxysm plasmas (PP) and normal human plasmas (NHP) were used as positive and negative controls. The bars indicate the median values of the data series together with the interquartile range (25%–75%).

Pre-incubation of PP with anti-cytokine antibodies viz. IL-1α, IL-1β, IL-2, IL-3, IL-4, IL-6, IL-10, IFN γ, TNF-α and GM-CSF, reduced the PP-induced leukocyte aggregation by 45% – 76% (Figure. 3). However, reconstitution of the cytokine-depleted plasmas with the recombinant human cytokines caused over 50% recovery of paroxysm plasma-induced cell aggregation only in the case of TNF-α, GM-CSF, IL-6 or IL-10 (Figure 3) suggesting that these are the principal cytokines involved in inducing leukocyte aggregation. However, TNF-α, GM-CSF, IL-6 and IL-10 induced little leukocyte aggregation, either when added to normal human plasma singly (data not shown) or in combination (Table 1: Expt.4 cf Expt.1) or when they were added to post-paroxysm plasma (Table 1: Expt.8 cf Expt.3).

These findings suggested that, in addition to these cytokines, further active factor(s), which are absent from both normal human plasma and post paroxysm plasma, must be present in paroxysm plasma to induce leukocyte aggregation. It was postulated that these additional factors could be parasitic in origin. To test this hypothesis, paroxysm plasmas were pre-incubated with immune serum which had been raised in rabbits against *P. vivax *blood stage schizonts. This treatment greatly reduced (by 75%) the paroxysm plasma-induced cell aggregation (Table 1: Expt.13 cf Expt.2; p < 0.05). Similarly, cell aggregate-inducing activity of paroxysm plasma was reduced when pre-incubated with convalescent sera collected following *P. vivax *infections in semi-immune individuals (Table 1: Expt.15; p < 0.05). These results suggest a likely role of parasite-derived molecules in this phenomenon.

Paroxysm plasma-induced cell aggregation was also inhibited, although to a lesser extent (by 46% only), by immune rabbit sera raised against *P. falciparum *blood stage schizonts (Table 1: Expt.14 cf Expt.2; p < 0.05). This suggests that the active molecules of putative parasite origin have species cross-reactive components. However, in view of the lesser effect of the anti-*P. falciparum *immune serum compared to that of antiserum raised against *P. vivax *schizont extracts (p < 0.05), species-specific active moieties could also be involved.

### Induction of leukocyte aggregation *in vitro *with extracts of *P. vivax *blood stage schizonts

The hypothesis that moieties of parasite origin might be involved in mediating the *in vitro *aggregation of white cells was further investigated. It was found that a strong leukocyte aggregation could, indeed, be induced by the addition of the combination of freeze-thawed extracts of *P. vivax *schizonts (but not extracts of uninfected human RBCs) and the cytokines rhu-TNF-α, GM-CSF, IL-6 and IL-10 to normal human plasma (Table 1: Expt.6 cf, Expts.7 & 1;) or to post paroxysm plasma (Table 1: Expt.10 cf Expt.3). By contrast, neither the cytokines nor the *P. vivax *schizont extract had any significant level of activity when added either to normal human plasmas on their own (Table 1: Expts. 4 & 5) or to post paroxysm plasma (Table 1: Expts.8 & 9). Moreover, the bacterial endotoxin, lipopolysaccharide (LPS), failed to substitute for the *P. vivax *schizont extracts in mediating these effects (Table 1: Expts.11 & 12).

These results are consistent with the hypothesis that the active, non-cytokine, factors in paroxysm plasma from non-immune patients who presented with acute *P. vivax *infections, which are involved in mediating white cell aggregation *in vitro*, are products of the parasites themselves and are specific to *Plasmodium *if not to *P. vivax *itself (see previous section of results). They cannot, for example be substituted for by a bacterial endotoxin such as LPS.

### Effect of heating, filtration and ultra-centrifugation of paroxysm plasma from acute *P. vivax *– infected patients on the induction of leukocyte aggregation

#### Heating

**Figure 4:**
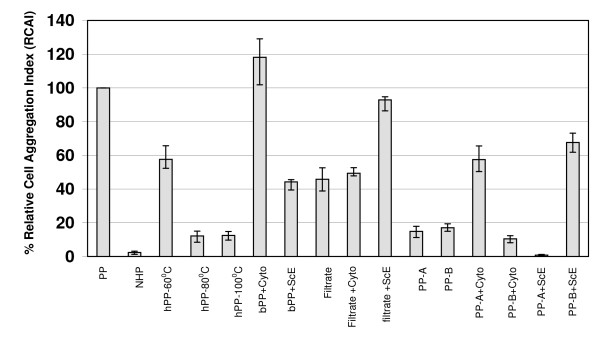
**Characterization of biologically active factor(s) in *Plasmodium vivax *paroxysm plasma**. Paroxysm plasmas from *P. vivax *patients (n = 6) were tested in the white blood cell aggregation assay before and after treatment of the plasmas by heating, filtration or centrifugation and with or without supplementation with recombinant human cytokines or freeze-thawed parasite schizont extracts. The bars indicate the median values of the data series together with the interquartile range (25%–75%). PP: Paroxysm plasma (untreated); NHP: normal human plasma (untreated); hPP: paroxysm plasma pre-heated at the indicated temperature; bPP: paroxysm plasma pre-treated at 100°C; PP-A: top layer of centrifuged paroxysm plasma; PP-B: bottom layer of centrifuged paroxysm plasma; Cyto: cytokines (TNF-∝, GM-CSF, IL-6 and IL-10); ScE: freeze-thawed *P. vivax *schizont extract.

Paroxysm plasma heated in a water bath at, or above, 80°C almost completely lost its ability subsequently to induce leukocyte aggregation. Thus, the CAI of paroxysm plasma heated at 100°C (hPP-100°C) was significantly lower than that of untreated paroxysm plasma (p < 0.05). The effect was completely restored, however, by addition of the "effective" cytokine combination (rhu-TNFα, GM-CSF, IL-6 and IL-10, as identified in this study) to heat-inactivated paroxysm plasma (bPP + Cyto) with CAI of bPP + Cyto being significantly higher when compared to that of heat-inactivated paroxysm plasma on its own (hPP-100°C); p < 0.05. By contrast, addition of *P. vivax *schizont extracts to the heat-inactivated paroxysm plasma (bPP + ScE) had little capacity to restore the white cell aggregating activity (p > 0.05) (Figure 4).

These results demonstrate that there is a heat stable (withstanding temperature up to 100°C) activity in paroxysm plasma obtained from acute *P. vivax*-infected patients, which is dependent upon the presence of the heat labile "effective" cytokine combination to mediate leukocyte aggregation. The material mediating the heat stable activity is likely to be of parasite origin.

#### Filtration

Filtration of paroxysm plasma (0.45 μm filters, Milipore-Sartorius AG), reduced, but did not eliminate, its ability to induce white cell aggregation (Figure 4) indicating that some of the active components in paroxysm plasma are in particulate form. Addition of the "effective" cytokines to the filtered plasmas did not restore the activity while addition of a *P. vivax *schizont extract did (Figure 4). This suggests that the activity reduced by filtration is likely to be parasite in origin.

#### Centrifugation

High-speed centrifugation of paroxysm plasma, or of normal human plasma as a control, produced two layers. The upper layer was thin and opaque but was wider and more distinct in paroxysm plasma compared to normal human plasma; this layer tested strongly positive for lipids in the Sudan III test. The lower layer was a clear solution both in paroxysm plasma and in normal human plasma and was negative for lipids by the Sudan III test. Compared to the original paroxysm plasmas from which they were derived, the material in each layer had greatly reduced ability to induce white cell aggregation (Figure 4). However, the activity was largely restored to the material in the top layer by the addition of cytokines (but not of *P. vivax *schizont extracts); activity was restored to the material in the bottom layer by the addition of *P. vivax *schizont extracts (but not cytokines) (Figure 4).

These results indicate that the active factors of putative parasite origin in paroxysm plasmas from non-immune patients infected with P. vivax infections were concentrated in the top, lipid-rich, layer and the cytokines were in the bottom, lipid-free, layer of the plasmas following high-speed centrifugation.

### The white blood cell-aggregating activity of different lipid-containing fractions of paroxysm plasma and *P. vivax *schizont extracts

Except for one fraction (P1); (Table 2; Exp 1), the other four lipid fractions extracted from heat-treated paroxysm plasma (P2 to P5) and freeze-thawed extracts of *P. vivax *schizonts (S1 to S5) showed very little effect when tested on their own in the cell aggregation assay (Table 2: Expts. 2–5 and 11–15 respectively). However, when reconstituted with the recombinant human cytokines rh TNF α, rh GM-CSF, rh IL-10 and rh IL-6, two of the five lipid fractions, one containing cholesterol/triglycerides (P1 and S1) and the other which contains phospholipids (P4 and S4) induced significant levels of cell aggregation (Table 2: Expts. 6 & 16 and 9 & 19 respectively).

**Table 2:** Leukocyte aggregation-inducing effects of lipid fractions of *Plasmodium vivax *paroxysm plasma and of *P. vivax *schizont extracts with or without supplementation with recombinant human cytokines (TNFα, GM-CSF, IL-6 and IL-10).

**Expt. No.**	**Sample**	**% R CAI (Median)**	**25%**	**75%**
1	P1	26.30	20.10	30.60
2	P2	4.50	3.40	8.60
3	P3	4.20	3.50	6.40
4	P4	8.50	6.80	11.80
5	P5	1.00	0.20	1.30
6	P1 + Cytokines	38.20	34.20	46.50
7	P2 + Cytokines	8.70	5.60	10.90
8	P3 + Cytokines	8.60	4.60	12.40
9	P4 + Cytokines	24.60	22.50	31.70
10	P5 + Cytokines	1.20	0.50	3.60
11	S1	3.1	1.4	6.4
12	S2	2.5	1.3	5.9
13	S3	2.9	1.2	7.4
14	S4	14.3	12.3	18.6
15	S5	1	0.2	2.4
16	S1 + Cytokines	10.4	7.6	13.9
17	S2 + Cytokines	3.8	2.5	6.4
18	S3 + Cytokines	2.7	1.9	6.5
19	S4 + Cytokines	26.2	21.9	30.4
20	S5 + Cytokines	1.1	0.3	2.1

% RCAI: % Relative Cell Aggregation Index (median)

25%–75%: 25th and 75th percentiles

Different fractions of *P. vivax *schizont extracts (S1 – acetone fraction, S2 – ethanol fraction, S3 – petroleum ether fraction, S4 – diethyl ether fraction and S5 – remaining fraction ) and similar fractions of paroxysm plasma ( P1 – acetone fraction, P2 – ethanol fraction, P3 – petroleum ether fraction, P4 – diethyl ether fraction and P5 – remaining fraction ) were tested for their ability to induce cell aggregation with or without supplementation with recombinant human cytokines (TNFα, GM-CSF, IL-6 and IL-10).

The table provides the median values together with the interquartile range (25%–75%).

In the lipid fractions of the *P. vivax *schizont extract, the more prominent effect was seen in the phospholipid-containing fraction (S4) (Table 2: Expt. 19). By contrast, the greatest activity from the paroxysm plasma was in the cholesterol/triglyceride fraction (P1) (Table 2: Expt.6). Moreover, the cell aggregating activity in this fraction was quite high even without addition of cytokines, which added relatively little to its activity (Table 2: Expts 1 & 6). This suggests that the lipids in this fraction may be competent to induce the necessary cytokines themselves, whereas those in other fractions of paroxysm plasma, and those from the schizont extract, are not. The results indicate that the active parasite-derived materials in the paroxysm plasmas and in those in the schizont extracts differ from each other either quantitatively or qualitatively.

## Discussion

The present study describes an *in vitro *phenomenon, the aggregation of white blood cells, which is mediated by the activity of factors present in plasma taken at the time of a paroxysm of *P. vivax *malaria. In the presence of paroxysm plasma from non-immune *P. vivax*-infected patients, aggregates were formed by white blood cells collected from healthy uninfected and malariologically-naive individuals. Similar aggregates were also seen when cells were obtained from *P. vivax*-infected patients and similarly incubated with paroxysm plasma. Although the biological relevance of such aggregate formation remains speculative, it might be a scavenging mechanism used by the host to get rid of the parasite debris released following schizont rupture [13].

Leukocyte activation associated with fever episodes has been shown in *P. vivax *malaria [14]. Peripheral leukopenia in *P. vivax *infections has been observed to be at its maximum during paroxysms (authors' unpublished observations). Pulmonary accumulation and sequestration of neutrophils and monocytes have been seen in murine models [15,16]. Furthermore, pulmonary oedema has been reported in *P. vivax *malaria [17], which may be due to accumulation of leukocytes. In *P. falciparum *malaria, cyto-adherent infected erythrocytes that bind to leukocytes enhance antibody-independent phagocytosis and induce cellular aggregation [18]. Pancytopaenia, especially thrombocytopaenia is common in malaria patients [19,20]. These findings provide indirect evidence as to the possible *in vivo *significance of such aggregate formation in malarial infections.

The active plasma factors, which mediated the cell aggregation included heat-labile substances consisting of mainly the monocyte-derived cytokines TNFα, GM-CSF and IL-6 and a T cell-derived cytokine IL-10. However, the activity of these cytokines in mediating leukocyte aggregation was absolutely dependent upon the simultaneous presence of heat-stable, and presumably parasite-derived, material released into the plasma at the time of a *P. vivax *paroxysm. It has been reported that aggregation of neutrophils may develop in EDTA – anti-coagulated blood [21]. However, in the present study EDTA-dependent aggregation of white blood cells can be excluded based on the experiments carried out using normal human plasma samples obtained from healthy individuals into similar EDTA concentrations, which did not show aggregation. Moreover, immune serum raised against *P. vivax *parasites could reverse this effect.

Ever since the description, at the end of the 19^th ^century, of the synchronous rupture of blood stage schizonts during human malarial infections and the association of these events with the periodic fevers of malaria, it has been clear that parasite products released into the plasma by the rupturing schizonts must be crucial to the initiation of a malarial paroxysm [22]. Evidence for the existence of malaria paroxysm-associated pyrogens, or "malaria toxins" as they were known, was provided by demonstrating that plasma from an individual undergoing a paroxysm of *P. vivax *malaria could rapidly induce equivalent symptoms when the plasma was injected into the circulation of a healthy volunteer [23]. Thus the concept of a malaria toxin is not novel [2,13,22-26]. It is now evident that the mediators of a malarial paroxysm, which include the parasite products, or "toxins", themselves, are involved in interactions with and between circulating white blood cells.

In addition to cytokines, the activated white blood cells release free radicals and other active molecular intermediates [27,28]. These mediators of inflammatory and pathogenic effects and also anti-parasitic effects [29-33] are speculated to help control parasite densities at the initial phase of a malarial infection. Inhibition of the maturation of *P. falciparum *schizonts *in vitro *by paroxysm serum from a case of *P. vivax *malaria has been demonstrated by other workers [33]. Similar anti-parasitic effect of paroxysm plasma from acute *P. vivax*-infected patients has been demonstrated to induce suppression of infectivity of malarial gametocytes to mosquitoes [1]. Like the cell aggregation effect described here, paroxysm-associated inactivation of gametocytes is mediated by cytokines and parasite products released at the time of the paroxysm [1,2,13]. In both the cell aggregation and the gametocyte inactivation phenomena, however, the parasite materials and moieties involved remain to be identified.

This paper describes the initial attempts made at characterization of the nature of these active parasite products. It is shown here that the heat-stable, cell-aggregating activity present in the paroxysm plasmas has the following properties. It is present in, and only in, plasmas taken at the time of the acute symptoms of a *P. vivax *paroxysm. No equivalent activity is present either before the acute symptoms begin or more than one or two hours after they cease. No equivalent activity is found in association with non-malarious fevers. The white cell-aggregating activity can be effectively substituted by extracts of schizonts of either *P. vivax *or *P. falciparum *when these are added to normal human plasmas in the presence of the cytokine combination identified above. Moreover, the activity in the paroxysm plasmas from acute *P. vivax*-infected patients is neutralised in the presence of immune sera raised against extracts of schizonts of either *P. vivax *or *P. falciparum*. The activity is more effectively neutralised by immune serum against *P. vivax *than that against *P. falciparum*, and is supportive evidence of parasite species-specificity.

These properties of the putative parasite-derived activity in *P. vivax *paroxysm plasma in relation to white cell aggregation are indistinguishable from those identified in relation to paroxysm plasma-mediated gametocyte inactivation [1,2,13,34]. The difference between the mediators involved in these two paroxysm-associated phenomena lies in the combination of cytokines involved. Thus the necessary and sufficient cytokines involved in gametocyte inactivation are the monocyte-derived cytokines TNFα, GM-CSF and the T cell derived IL-2; those involved in the white cell aggregation are principally the monocyte-derived TNFα, GM-CSF and IL-6 and the mainly T cell derived cytokine IL-10. In both phenomena the immediate presence of monocytes was shown to be essential. On the other hand, the depletion of T lymphocytes did not affect cell aggregation. This does not, however, exclude a role for T cells at an earlier stage in the events of a paroxysm as, for example, during the induction of other cytokines involved in the process. This is probably because, the T cell-derived cytokines having already been induced by the time of the active paroxysm. Therefore, the continued immediate presence of the T cells is no longer essential for either gametocyte inactivation or white cell aggregation to take place. Though, T cells strongly respond to phosphoantigens from *Plasmodium *parasites [35], prolonged exposure to *P. vivax *malaria infections in endemic areas are known to cause immuno-suppression of human T cells [36].

The cell aggregation phenomenon was used as an assay to explore the physical and chemical nature of the heat stable, and presumably parasite-derived, activity in the paroxysm plasmas from acute *P. vivax*-infected patients. Results indicate a prominent role of cholesterol and triglycerides containing fraction of paroxysm plasma in mediating cell aggregation even in the absence of added cytokines. Only one other fraction gave significant activity. This was the phospholipid fraction and this activity was largely dependent upon the presence of the added cytokines. Equivalent lipidic fractions were made from extracts of schizonts of *P. vivax *but significant, and largely cytokine-dependent, activity was found only in the phospholipid fraction. Interestingly the characteristics of this fraction were congruent with those of the malaria parasite-derived TNF-inducing GPI moieties studied by other workers [37-43]. GPI surface-anchored antigens including MSP-1 have been viewed as potential candidates for vaccine development [44,45]. Studies in other laboratories have demonstrated lipogenesis-inducing activity in the lipid fractions of boiled supernatant of *P. falciparum *cultures [46] with subsets of T lymphocytes being affected at an early stage during paroxysms of non-endemic malaria infections [35].

## Conclusion

This study identifies leukocyte aggregation as a phenomenon associated with paroxysms in *P. vivax *infections. This phenomenon is mediated by plasma factors including host-derived cytokines and lipids of putative parasite origin. Most of the parasite-derived activity lies in the cholesterol/triglyceride and phospholipid fractions of paroxysm plasma and especially in the former fraction. Activity in this cholesterol/triglyceride-containing fraction is poorly represented in extracts of *P. vivax *schizonts prepared *in vitro*. Therefore, only *P. vivax *paroxysm plasma itself, and not artificial extracts of parasite material prepared *in vitro*, represent an adequate source of the biologically active parasite-derived mediators present during such a paroxysm. The characteristics of the phospholipid fraction in paroxysm plasma are congruent with those of the malaria parasite-derived, TNF-inducing GPI moieties described by other workers. The more active cholesterol/triglyceride(s), on the other hand, represent a new class of biologically active lipid associated with the paroxysm of *P. vivax *malaria.

In future investigations, it is planned to explore further the chemical characterisation of active parasite-derived mediators associated with the paroxysm of *P. vivax *malaria using *P. vivax *paroxysm plasma itself as the source of the active moieties.

## Authors' contributions

NK conceptualized and designed the study, interpreted the data and drafted the manuscript. DW carried out the laboratory experiments and data tabulation. VC provided advice on design, analysis and interpretation of biochemical characterization experiments. RC gave considerable intellectual input in analyzing the results and helped draft the manuscript and KM made contributions for interpretation of data and drafting the manuscript. All authors have given final approval of the version to be published. The research work was carried out at Malaria Research Unit, Faculty of Medicine, University of Colombo, Sri Lanka.
